# Organosolv Lignin as a Green Sizing Agent for Thermoformed Pulp Products

**DOI:** 10.1021/acsomega.2c05416

**Published:** 2022-12-08

**Authors:** Mihaela Tanase-Opedal, Jost Ruwoldt

**Affiliations:** RISE PFI AS, Høgskoleringen 6B, 7491Trondheim, Norway

## Abstract

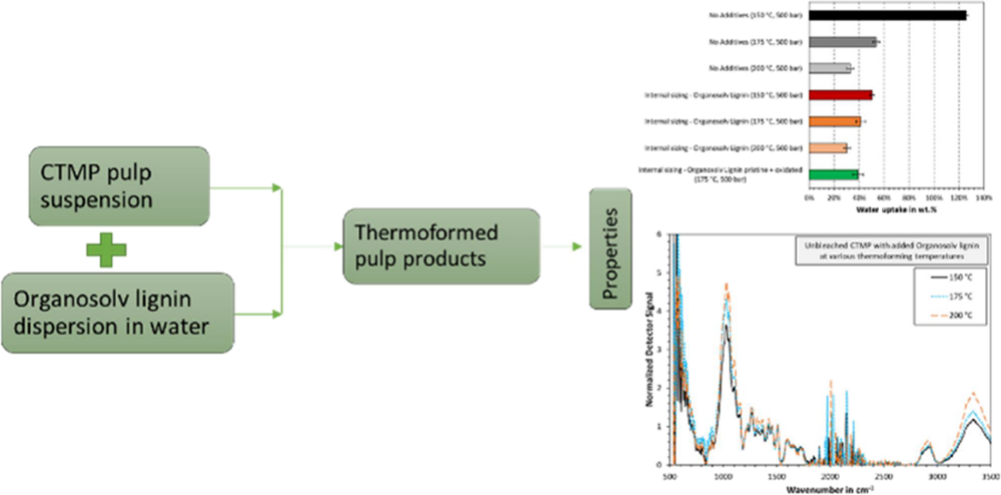

The purpose of this study was to investigate the use of organosolv lignin as a sizing agent for thermoformed pulp products as a sustainable material with improved water resistance. For this purpose, an in-house-produced organosolv lignin from softwood (Norway Spruce) was mixed with bleached and unbleached chemi-thermomechanical pulp fibers. In addition, the isolated organosolv lignin was characterized by ATR–FTIR spectroscopy, size-exclusion chromatography, and thermogravimetric analysis. The analysis showed that organosolv lignin was of a high purity and practically ash-free, exhibiting low molecular weight, a glass transition temperature below the thermoforming temperature, and a high content of phenolic OH groups. The mechanical properties and water resistance of the organosolv lignin-sized thermoformed pulp materials were measured. A small decrease in strength and an increase in stiffness and density were observed for the lignin-sized thermoformed materials compared to the reference, that is, unsized materials. The addition of organosolv lignin decreased the wettability and swelling of the thermoformed product. These results are due to the distribution of organosolv lignin on the surface, filling in the pores and cavities, and providing a tighter fit within the thermoformed materials. In conclusion, the results from our study encourage the use of organosolv lignin as a sizing additive to thermoformed products, which can improve the water resistance to use it in sustainable packaging applications.

## Introduction

In recent years, research on biomass valorization has focused on biorefinery processes that yield fermentable sugars for bioethanol production, while lignin has been collected as a low-value byproduct and used for cogeneration of heat and electricity. However, from the techno-economic perspective, the success of future biorefineries is highly dependent on lignin valorization.^1,2^ The interest for developing lignin-based bioproducts, including lignin-based chemicals and materials, has intensified in the past few years. This is due to several points: (1) environmental concerns and depletion of fossil-based resources and (2) the increasing availability of lignin as a renewable resource from biorefinery side streams.^3^ Lignin incorporation in biopolymer materials, such as filler in thermoplastics or pseudo-monomeric additive for bioplastic synthesis, has gained attention due to the strong mechanical reinforcement ability, antioxidant and antimicrobial properties, thermoplastic properties, and reduced wettability.^4−12^ However, success has so far been limited due to the lignin’s challenging processability, high molecular weight, amorphous structure, low reactivity, the intrinsic heterogeneity of lignin, and variations in composition and structure depending on the raw material and the extraction method.

Organosolv lignin obtained after organosolv fractionation tends to exhibit low molecular weight and polydispersity compared to other technical lignins due to the higher degree of depolymerization and a lower propensity for condensation reactions.^13,14^ In addition, organosolv lignin is sulfur-free with a very low ash content.^15^ Organosolv fractionation of lignocellulosic biomass is carried out at high temperatures and high pressures, with or without the addition of a catalyst.^16^ Moreover, the degree of delignification and the selectivity of the lignin extraction process depend on the operating conditions applied, especially the type of organic solvent and its concentration.^17^ Solvents are designed to reduce the reaction severity, correlated directly with the degradation and condensation of hemicellulose and lignin during the process. Sustainability and environmental impact of the solvent are important.^18^ Organosolv fractionation is described in the literature as a promising strategy for valorization of lignocellulosic biomass, which could facilitate the transition to improve utilization of renewable feedstocks.^19^

The concept of using fiber materials and polymers from the biomass is interesting since the material would be completely based on renewable resources. One example within industrial materials is molded pulp, which is commonly used for packaging.^20^ Molded fiber products are manufactured from various chemical pulps and chemi-thermomechanical pulps (CTMPs), which are pressed into the desired shape in a molding machine. The molded fiber technology enables manufacturing of products that can replace plastic, such as single-use food packaging products. Traditionally, paperboard packaging materials are coated with synthetic polymers that enhance their resistance to water, moisture, grease, oxygen, and odor.^21^ It has been shown that the introduction of biopolymers as alternatives to petroleum-based plastics potentially reduces carbon dioxide emissions by 30–70%,^22^ induces hydrophobicity, and may promote interfiber adhesion and mechanical properties.^23^ Moreover, lignin and its blends have been reported to provide both gas and UV light barrier properties, and they work as an antimicrobial coating.^24−26^ The hot-pressing technique used in molded pulp shows that it is possible to produce a material with low porosity and much improved mechanical properties compared with the existing fiber materials.^27^ Furthermore, Joelsson et al. confirmed that increased temperature combined with sufficient pressure enabled permanent densification by softening of lignin, producing very high tensile strength. The mechanical strength of the molded fiber products is believed to result from hydrogen bonding between the fibers, condensation reactions of lignin, and partial degradation of hemicellulose.^28^ Literature data showed that 6–17% residual lignin in the fibers may distinctly contribute to the strength, stiffness, and water resistance of the molded pulp products.^29^

The literature discussed above suggests that lignin can be used as a sizing additive for molded fiber products, which can enhance the water-repellent properties while improving the mechanical properties. In our study, we therefore tested in-house-produced organosolv lignin as a sizing additive to prepare thermoformed pulp products. The mechanical properties and hydrophobicity of these materials were analyzed and discussed in this study.

## Materials and Methods

### Materials

Bleached and unbleached CTMPs with a Canadian standard freeness (CSF) of 450 and an ISO brightness of 80 and 60, respectively, were kindly provided by MM Karton FollaCell AS. Acetone (SA quality, >99.7%) was obtained from ROMIL. Distilled water was used, if not otherwise specified.

The organosolv fractionation of Norway spruce was carried out in an autoclave reactor system from TOP industries, France. For more details on the autoclave, see ref (30). In short, 50 g of dried wood chips was charged into the autoclave and treated with aqueous acetone (50:50, w/w) and sulfuric acid as a catalyst (1% w/w, per dry wood weight) at 195 °C for 30 min. The liquid/solid ratio was 7.5:1. After the cooking time ended, the autoclave was rapidly cooled down in cold water to quench the reaction. The solids were separated from the liquid by filtration. The filtrate was further used to precipitate the organosolv lignin by diluting it three times with deionized water, filtering through a Whatman filter paper, and thoroughly washing with deionized water. The isolated organosolv lignin was dried at room temperature.

### Thermoforming of Material Specimens

All material specimens were made from pulp suspensions with 2.7–3 g/L consistency. Next, 1.6 g_DM_ of organosolv lignin was first dispersed in 500 mL of water, which yielded 40 wt % added lignin per dry fiber mass. After blending the lignin and pulp suspensions, 200 ppm of a cationic flocculant (PCB 20, Solenis Norway) was added to facilitate binding of the particles to the fibers and hence a more homogeneous distribution in the final product. The suspension was finally vacuum-filtrated into the mold. The same procedure was used as in our previous study.^12^ In short, the wet material was first pressed while increasing the temperature to 80 °C for 5 min. Afterward, the mold was heated to 155 °C for 10 min followed by pressing at 180 bar isothermally at 155 °C for 5 min. All specimens were weighed, and the thickness was measured to calculate the density.

### Handsheet Preparation

The sample composition and pulp suspensions were the same as for the thermoformed material explained above. Handsheets were prepared according to the ISO 5269-1:2005 “Rapid-Köthen method.” Two procedures were adapted for the preparation of handsheets with added lignin:

1) In the first implementation, the wet handsheets were cold-pressed between laboratory paper at 20 bar and subsequently air-dried to a dry matter content of approximately 90%. The dried sheets were then pressed between metal plates at 45 bar and 150 °C.
2) In the second implementation, the wet handsheets were pressed between laboratory paper at 500 bar and 90 °C for 3 min. The temperature was then raised to the target temperature (150, 175, or 200 °C) over a duration of 30 min. Afterward, the sheets were pressed at 500 bar for 5 min while maintaining the target temperature.

The composition of handsheets was the same as that of the thermoformed material specimens. The organosolv lignin was added to the fiber suspension as (1) an internal sizing agent where 1.6 g of organosolv lignin (dry matter weight) was added to the fiber suspension prior to sheet formation; (2) an impregnation agent where the impregnation of sheets was done by dissolving 20 wt % lignin in dimethyl sulfoxide (DMSO), distributing the lignin solution on the sheet with a bar coater, air drying, and finally hot pressing; and (3) as a coating agent by dissolving 10 wt % lignin and 10 wt % cationic starch (Perlbond 930, Lyckeby Stärkelsen AB, Sweden) in DMSO, coating with a bar coater at a gap width of 300 μm, air drying, followed by hot pressing.

In addition, oxidation of organosolv lignin was carried out according to the optimized procedure of He et al.’s study.^31^ In this implementation, lignin was dissolved in an aqueous NaOH solution and oxidized with H_2_O_2_ at 80 °C for 2 h, using a molar ratio of 0.77 and 2.85 for NaOH/H_2_O_2_ and H_2_O_2_/lignin, respectively. When preparing the samples, of the total 1.6 g of organosolv lignin added to the handsheet, 0.6 g was replaced with oxidized organosolv lignin.

## Analytical Procedures

### Characterization of Organosolv Lignin

The dry matter content was determined after oven drying at 105 °C for at least 3 h. The ash content was measured according to ISO 1762:2019, that is, by incineration at up to 525 °C. Acid-insoluble lignin (Klason lignin) and acid-soluble lignin were determined based on TAPPI T 222 om-02. The procedure was modified in which the biomass was first digested in concentrated sulfuric acid (72% H_2_SO_4_) at 30 °C for 1 h and then in diluted sulfuric acid (4% H_2_SO_4_) for 1 h at up to 121 °C. Acid-insoluble lignin was measured gravimetrically, whereas acid-soluble lignin was determined via UV absorbance at 205 nm. The number of phenolic hydroxyl groups was measured by non-aqueous potentiometric titration using a modified version of Lin and Dence.^32^ In short, 0.15 g of lignin and 0.1 g of internal standard (4-hydroxybenzoic acid) were dissolved in 60 mL of DMSO and titrated with 0.1 N *tetra*-*n*-butylammonium hydroxide while measuring the electric potential. The number of carboxylic acid and phenolic hydroxyl groups, respectively, was determined from the inflection point of the titration curve.

### Attenuated Total Reflectance–Fourier Transform Infrared (ATR–FTIR) Spectroscopy

ATR–FTIR spectroscopy was performed using a PerkinElmer Spectrum 3 with a universal ATR sampling accessory. The air-dried lignin samples were pressed onto the FTIR prism using in-software correction for residual moisture. For each sample, 32 runs were performed and averaged using a step rate of 4 cm^–1^. Graphs were baseline-corrected and normalized via the aromatic skeletal vibration band at 1510–1505 cm^–1^ or the C–H stretching band at 2930–2940 cm^–1^.

### Thermogravimetric Analysis Coupled with Differential Scanning Calorimetry (TGA–DSC)

TGA–DSC was conducted using a NETZSCH STA 449 F3 Jupiter using the TGA–DSC socket. A steady flow of nitrogen at 50 mL/min ensured an inert atmosphere. Each alumina crucible was loaded with 10.0 ± 0.1 mg of lignin. Heating was conducted first at a rate of 5 °C/min to 105 °C, followed by holding this temperature for 5 min. Afterward, the sample was cooled to 35 °C and finally heated at 5 °C/min to 250 °C for the actual measurement. This two-step approach ensured that the final measurement was conducted on virtually dry lignin as moisture reportedly affects the glass transition temperature. The DSC signal was further converted to the apparent heat capacity as this compensated for a nonuniform heating rate in the beginning of the program (<80 °C). The glass transition temperature was determined as described in our previous study.^12^ Here, the baseline and step change in apparent heat capacity were fitted by a straight line, computing the onset as the first and the glass transition temperature as the average of first and second intersections.

### Mechanical Testing

Mechanical testing of the formed material specimens was performed using a Zwick material tester. The specimens were first equilibrated at 22.0 °C and 50% relative humidity for at least 24 h. Tensile tests took place at an elongation rate of 5 mm/min. The ultimate force was converted to the ultimate stress by division through the cross-sectional area of the breakage section. Young’s modulus was determined from the instantaneous slope at 0.5% elongation.

### Microscopy

Top-down images were recorded using a Leica Wild M8 stereomicroscope fitted with a ProgRes SpeedXT Core 5 digital camera. Light transmission images were recorded using a Leica light microscope also fitted with a ProgRes SpeedXT Core 5 digital camera.

### Contact Angle

Contact angle measurements were conducted using a dynamic adsorption tester (DAT) 112 from Fibro Systems, the Netherlands. For this, the handsheets were first cut into long stripes and equilibrated at 22.0 °C and 50% relative humidity for at least 24 h. During each measurement, 3–4 μL of distilled water was dropped onto a new section of the test stripe while recording the droplet shape with a high-speed camera. Angle determination and data recording were performed using in-software. Two stripes were tested per sample with 10 measurements each, yielding a final of 20 measurements per sample.

### Water Uptake

Test pieces measuring 40 mm × 13 mm were cut out from the material specimens and weighed. The pieces were immersed in water for 30 min, wiped with laboratory paper, and weighed again to calculate the water uptake. Handsheets were cut into test stripes measuring 10 cm × 1.5 cm and immersed in water for 24 h. Afterward, the test stripes were wiped with laboratory paper and weighed again. In both cases, the water uptake *P*_water_ was calculated as the difference of wet *m*_wet_ and dry *m*_dry_ mass per dry mass, as shown in eq 1.
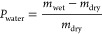
1

## Results and Discussion

### Lignin Characterization

The chemical and physical properties of lignin depend on the wood species, the pretreatment/pulping process, and the isolation process. The composition and analytical results of the organosolv lignin are listed in Table 1. The values for dry matter (DM) and ash content are realistic as a DM of 95% is frequently encountered for technical lignin and a low ash content is characteristic for organosolv pulping. Measurements of the acid-insoluble and acid-soluble lignins furthermore attest the production of a very pure sample as the total lignin content is close to 100%. This is confirmed by the measurements of phenolic hydroxyl groups using non-aqueous titration where 4.91 mmol/g is higher than existing lignin qualities on the market.^33,34^ Measurements of the molecular weight (*M*_w_) in addition confirm a high degree of depolymerization, which would result in the availability of the determined free phenolic hydroxyl groups. Literature results show that the severity of the organosolv fractionation method of the wood biomass has a high impact on lignin’s *M*_w_.^35^ At high severity, the condensation reactions occur that increase the sample dispersity toward higher *M*_w_ values. A lignin rich in G units is more susceptible to these changes.^36^ However, our results show that the organosolv lignin has a low molecular mass and abundance of phenolic OH groups. A lignin with a low molecular weight and abundance of phenolic OH groups has a strong natural antioxidant property,^37^ which is desired in packaging applications. Another advantage of using lignin with a low molecular weight is that this could facilitate condensation reactions of lignin during the molding process.^38^ As such, the produced organosolv lignin represents a promising renewable biopolymer that can be used to replace the fossil-based polymers in packaging applications.

**Table 1:** Analytical Data of Organosolv Lignin

DM content	wt %	94.91
ash content	wt %	0.28
acid-insoluble lignin	wt % per DM	98.7 ± 0.1
acid-soluble lignin	wt % per DM	1.4 ± 4.8
onset temperature of the glass transition event	°C	135.4 ± 0.1
glass transition temperature	°C	146.0 ± 0.1
phenolic hydroxyl groups	mmol/g	4.91
number average molecular weight (*M*_n_)	g/mol	1100
mass average molecular weight (*M*_w_)	g/mol	3300
polydispersity index (PI)		3.0

### Glass Transition Temperature

**Figure 1:**
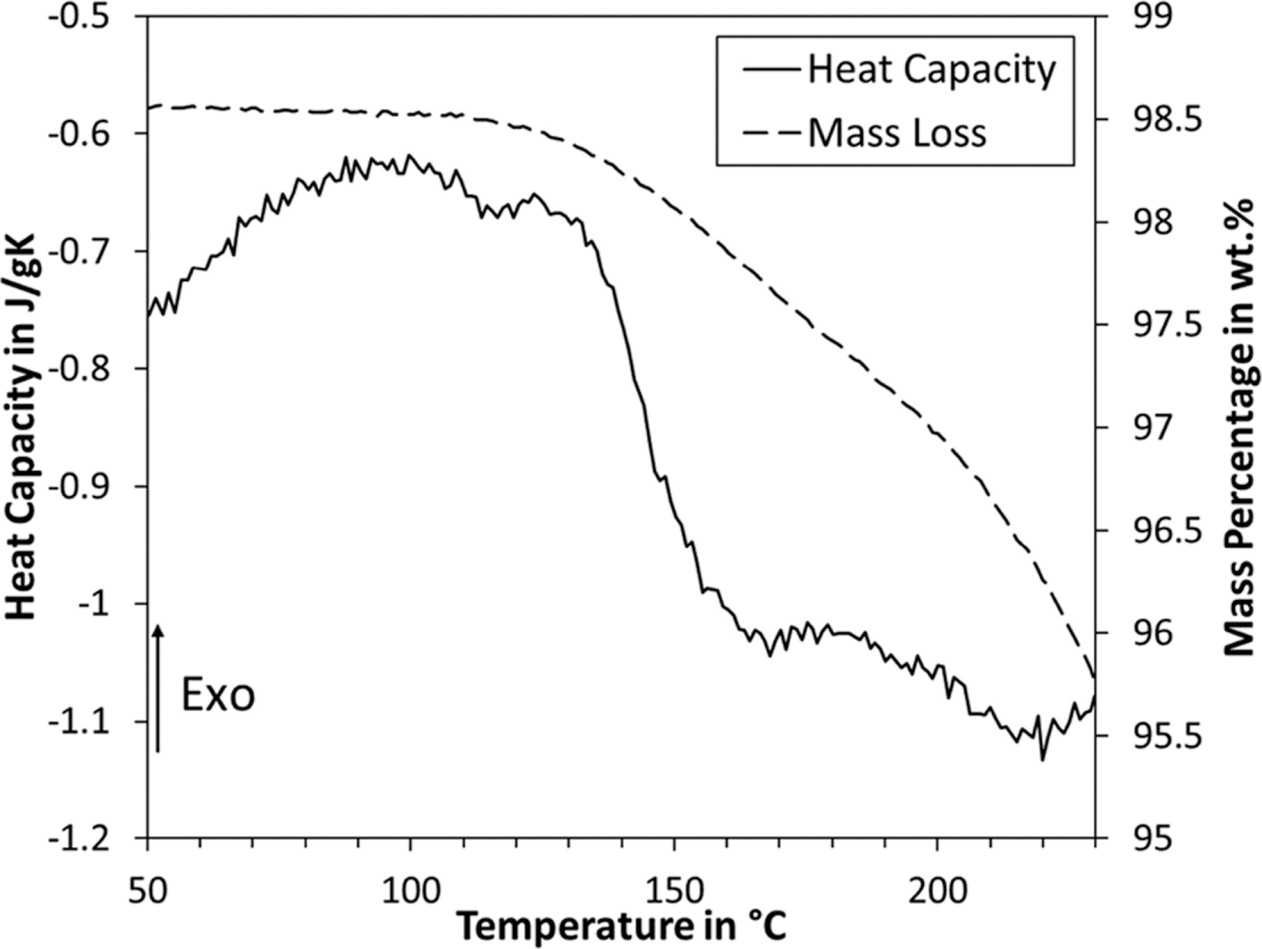
TGA and DSC signals of organosolv lignin. The data were average over two measurements.

The glass transition temperature is correlated with a change in the viscoelastic behavior of amorphous polymers. In addition, the glass transition temperature (*T*_g_) of lignin is strongly influenced by the water content and the presence of solvents. At temperatures below glass transition, viscoelastic materials are stiff and brittle, whereas the stiffness decreases in the transition region.^39^ The data obtained by TGA–DSC measurements are plotted in Figure 1. As can be seen, the TGA signal shows a constant value of 98.5% up to 110 °C. Afterward, the mass percentage steadily decreases likely because of the evaporation of volatiles. Above 200 °C, the mass percentage decreases at a higher slope, which can be attributed to the thermal decomposition of the sample. Despite the evaporation of volatiles, a clear step change in heat capacity is observed between 130 and 150 °C. The fitting procedure determined a *T*_g_ of 135.4 °C, as listed in Table 1. Drying at 105 °C before the measurement was most likely incomplete as the analytically determined DM was closer to 94.9%. It is hence evident that the measured *T*_g_ is lower than the real value; however, the underestimation is likely minor as the difference is only 2.6%. Our results are in accordance with the literature results where the *T*_g_ value depends on the pretreatment method and biomass type, and it ranged from 90 to 180 °C.^40,41^ A small decrease in apparent heat capacity can be noted above 200 °C, which agrees with the onset of thermal decomposition.

### FTIR

**Figure 2:**
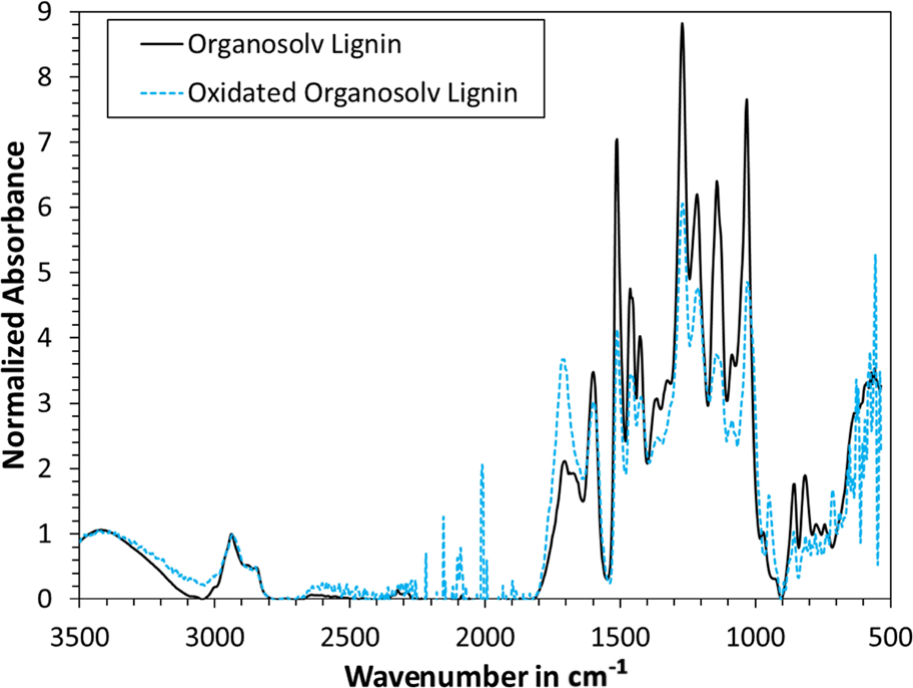
ATR–FTIR spectra of pristine and oxidized organosolv lignin. Each graph was baseline-corrected and normalized via the C–H stretching band at 2930–2940 cm^–1^.

The baseline-corrected FTIR spectra are plotted in Figure 2. The graphs were normalized using the C–H stretching band at 2930–2940 cm^–1^ as oxidation of lignin deteriorates the phenolic moieties, hence rendering the aromatic skeletal vibrations at 1505–1510 cm^–1^ unrepresentative. It is interesting to note that based on this normalization, the abundance of hydroxyl groups (O–H stretching band at 3412–3460 cm^–1^) remained unchanged. Reduction of the phenolic hydroxyl groups hence seems to be compensated by the generation of carboxylic groups, which may also contribute to the O–H stretching of this band. The peak at 1720 cm^–1^ is much more pronounced after oxidation, which is generally attributed to C=O stretching. Such intensification is evidence for the generation of new carboxyl groups as these moieties also contribute to the C=O stretching. It can, however, not be ruled out that also unconjugated ketones, carbonyls, and esters were generated in the process. Both the pristine and oxidized organosolv lignin show characteristic peaks at 1600 cm^–1^ (aromatic skeletal vibration plus C=O stretching), 1510 cm^–1^ (aromatic skeletal vibrations), 1460 cm^–1^ (C–H deformation), 1425 cm^–1^ (aromatic skeletal vibrations combined with C–H in-plane deformation), 1270 cm^–1^ (G ring plus C=O stretching), 1225 cm^–1^ (C–C, C–O, and C=O stretching), 1140 cm^–1^ (aromatic C–H in-plane deformation), and 1030 cm^–1^ (aromatic C–H in-plane deformation). All the latter are characteristic for G-type lignin as found in softwood, where the intensity is lower after oxidation, which is also in agreement with the degradation of phenolic moieties.

### Thermoformed Material Specimens

The addition of organosolv lignin to thermoformed fiber specimens was carried out as this allows testing the effect on mechanical properties. The observed trends agree with our previous findings, where the addition of technical lignin yielded an increase in stiffness and density.^12^ In addition, a decrease in water uptake was noted. This decrease is likely due to reduced wetting of the added lignin compared to cellulose. Densification can further decrease the mass transfer within the sample as voids are filled by the added lignin. The tensile strength was lower compared to the reference case without added lignin although we would have expected an increase in tensile strength (Table 2). Liu et al. reported that condensation reactions of lignin with furfural from degradation of hemicellulose gave an increase in tensile strength during the molding process. However, we do not know for sure that this hypothesis is applicable to our study. We believe that the lower tensile strength in our study can be related to (a) lignin showing a more brittle continuous phase, (b) the effect of added lignin on filtration speed, or (c) the thermoformed temperature is too low to see the effect of curing in the molding process. The filtration rate was lower after adding organosolv lignin as the fine particles can cause clogging and produce a denser filter cake. Slower filtration can result in the CTMP fibers attaining a less random distribution, which can result in worse tensile strength.

**Table 2:** Testing Results for Thermoformed Material Specimens with and without 40% Added Lignin^a^

fiber type	added lignin	tensile strength (MPa)	Young’s modulus (MPa)	density (kg/m^3)^	water uptake (wt %)	mass loss (wt %)
bleached CTMP	none	42.9 ± 2.4	2960 ± 78	939 ± 6	192 ± 11	2.0 ± 2.4
	organosolv	34.8 ± 3.3	3659 ± 154	1096 ± 13	123 ± 18	2.7 ± 0.5
unbleached CTMP	none	34.4 ± 3.2	2349 ± 35	915 ± 5	175 ± 18	1.1 ± 0.7
	organosolv	32.5 ± 2.9	2857 ± 188	1067 ± 12	103 ± 12	4.4 ± 0.5

a Each data point was averaged over four measurements.

**Figure 3:**
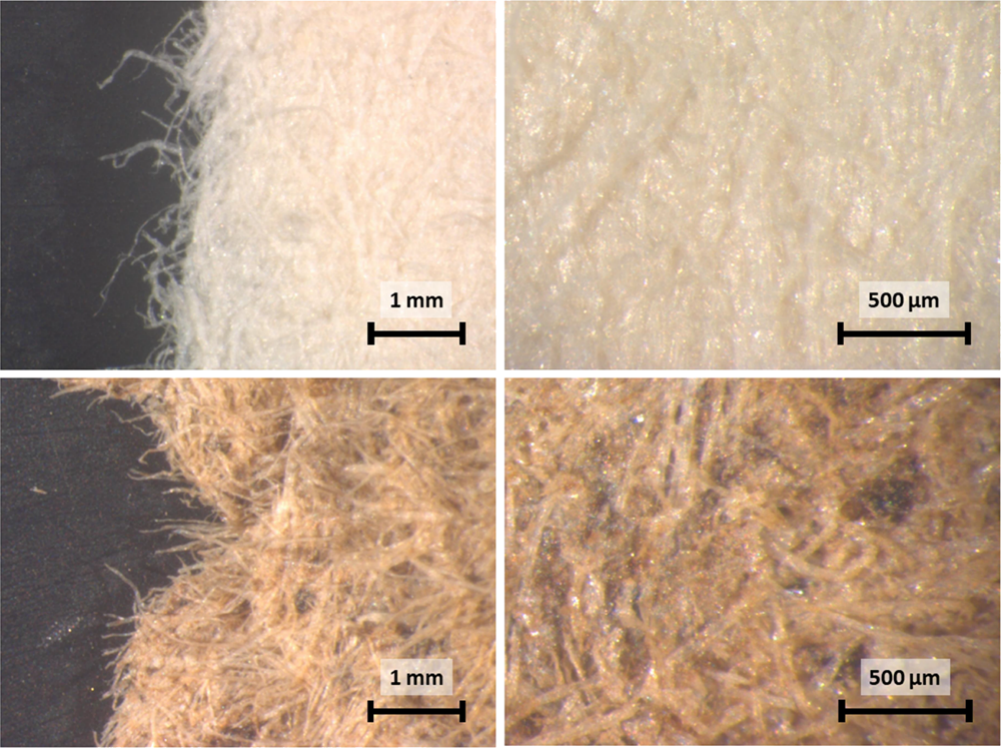
Stereomicroscope images of bleached CTMP (top) with added organosolv lignin (bottom) at 18× (left) and 50× magnification (right).

Microscope images of the thermoformed fiber specimens are shown in Figure 3. The added organosolv lignin was found within the entire material specimen as can be seen by the color of the fibers changing from white to light brown. Joelsson et al. found that when the fibers collapse, the lignin seemed to remain on the fiber surfaces and the contact area between the lignin-coated fibers increased. Dark spots show the existence of local concentration spikes. These are likely due to larger lignin particles or agglomerates which did not get dispersed during preparation. It is unlikely that the dark spots originate from filled in cavities as the spot diameter is considerably larger than the fiber width. The images on the left furthermore show the broken cross section as it was generated during destructive tensile testing. As can be seen, fibers are pointing outward in a brush-type manner. Addition of organosolv lignin did not yield visible tearing of fibers. In both cases, the breaking mechanism is hence pulling apart of the fiber network. This would suggest that cross-linking between the fibers and the added lignin was negligible, if it occurred at all.

### Thermoforming of Handsheets

Different application modes and thermoforming settings were explored for adding organosolv lignin to the handsheets. This was done to, among others, explore the effect of added lignin on wetting of the sheets. The main difference between handsheets and thermoformed material specimens is thickness as the handsheets were in the range of 0.1–0.3 mm, whereas the material specimens were in the range of 1.4–2 mm. An overview of the experiments is given in Table 3. As can be seen, the reference case without added lignin yielded a density of 307 kg/m^3^ when pressed at 150 °C and 45 bar. The density more than doubled after increasing the pressure to 500 bar, which suggests a strong impact of thermoforming pressure. At 500 bar, the density would further increase when elevating the temperature to 175 and 200 °C. The measured basis weight was constant when using the same input materials. A slight difference between bleached and unbleached CTMPs exists, which can be attributed to the fiber substrate. Adding organosolv lignin as an internal sizing agent showed a constant density with respect to temperature when pressing at 500 bar. Adding oxidized organosolv lignin yielded a lower density under the same setting; however, this difference is within the standard deviation. Thermoforming sheets with added lignin at 45 bar yielded approximately half the density compared to that at 500 bar. It is interesting to note that the sheets impregnated with organosolv lignin exhibit a density of 793 kg/m^3^, which is close to that of the sheets pressed at 500 bar. This observation agrees with the interpretation that the lignin fills cavities within the fiber matrix. The density of sheets coated with organosolv lignin and starch was the highest at 1159 kg/m^3^. It thus appears that the coating penetrated the fiber network even better than applying organosolv lignin alone. Synergies between the starch and added lignin are possible as the cationic charge of the starch may interact with the phenolic hydroxyl groups of lignin, which can attain a negative charge. Our observation agrees with the literature data where interactions have been shown between the hydroxyl, carbonyl, and ether groups of the starch and lignin components.^42,43^ Moreover, the addition of lignin in starch matrices modifies both the chemical and physical properties of the resulting product such as reducing the water solubility of starch in dry coatings.^42,44^ All in all, it appears that the lignin enhances the densification effect of thermoforming where a constant density was obtained at 850–860 kg/m^3^ using 500 bar pressure. Based on the basis weight, however, the amount of added lignin in the final product appears lower than the design input. A shortage of up to 25 g/m^2^ was noted, which would account for 25 wt % or less organosolv lignin per dry fiber weight. Considering an input design of 40 wt %, it thus appears that more organosolv lignin was lost in the handsheets than for the thermoformed material specimens. This is likely due to the distribution of fibers over a larger area and hence insufficient build-up of a filter cake.

**Table 3:** Application Mode and Thermoforming Settings of Handsheets^a^

fiber type	added lignin	application mode	thermoforming temperature (°C)	thermoforming pressure (bar)	density (kg/m^3^)	basis weight (g/m^2^)
bleached CTMP			150	45	307 ± 5	149.6 ± 2.5
bleached CTMP	organosolv lignin	internal sizing	150	45	346 ± 4	177.1 ± 0.5
bleached CTMP	organosolv lignin	impregnated	150	45	793 ± 81	177.7
bleached CTMP	organosolv lignin + cationic starch (50/50)	coated	150	45	1159 ± 35	217.2
unbleached CTMP			150	500	728 ± 12	126.6 ± 0.6
unbleached CTMP			175	500	789 ± 18	128.6 ± 0.6
unbleached CTMP			200	500	820 ± 6	134.1 ± 0.6
unbleached CTMP	organosolv lignin	internal sizing	150	500	853 ± 33	155.7 ± 0.7
unbleached CTMP	organosolv lignin	internal sizing	175	500	859 ± 14	150.4 ± 0.7
unbleached CTMP	organosolv lignin	internal sizing	200	500	851 ± 11	147.7 ± 0.7
unbleached CTMP	organosolv lignin (pristine + oxidized)	internal sizing	175	500	835 ± 27	158.3 ± 0.7

a Experimental errors are given as the standard deviation. Data for the reference case (bleached CTMP, 150 °C and 45 bar) were taken from our previous study.^12^

### Microscope Images

**Figure 4:**
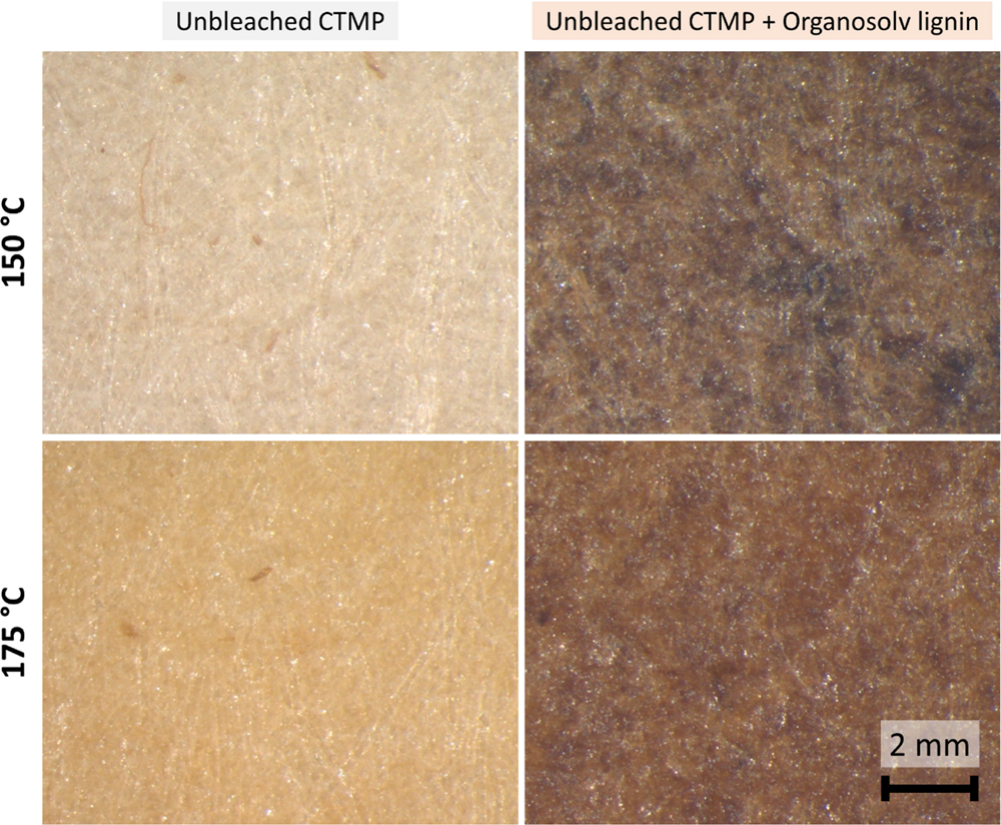
Top-down microscope images of unbleached CTMP (left) with organosolv lignin (right). The thermoforming pressure was 500 bar at a temperature of 150 °C (top) or 175 °C (bottom).

Top-down images of selected handsheets are depicted in Figure 4. Individual fibers show as long bright lines. Increasing the temperature also altered the fiber color from pale white to pale brown in cases where no lignin was added. Unbleached CTMP with added organosolv lignin appeared dark brown after pressing at 150 °C and red brown at 175 °C. Dark spots are visible at 150 °C, which may be attributed to the local concentration spikes of the added lignin. These could be due to inhomogeneities after adding the lignin as particles or due to filled in cavities. At 175 °C, no dark spots are visible, which would suggest that the distribution of added lignin was more homogeneous. The *T*_g_ of organosolv lignin was measured at 135 °C, which would entail that 150 °C should be sufficient to induce complete melting of the lignin. Nonetheless, it appears that a higher temperature is necessary. Moreover, it was noted that the surfaces thermoformed at 150 °C exhibited a higher roughness than the handsheets pressed at higher temperatures.

### Contact Angle

**Figure 5:**
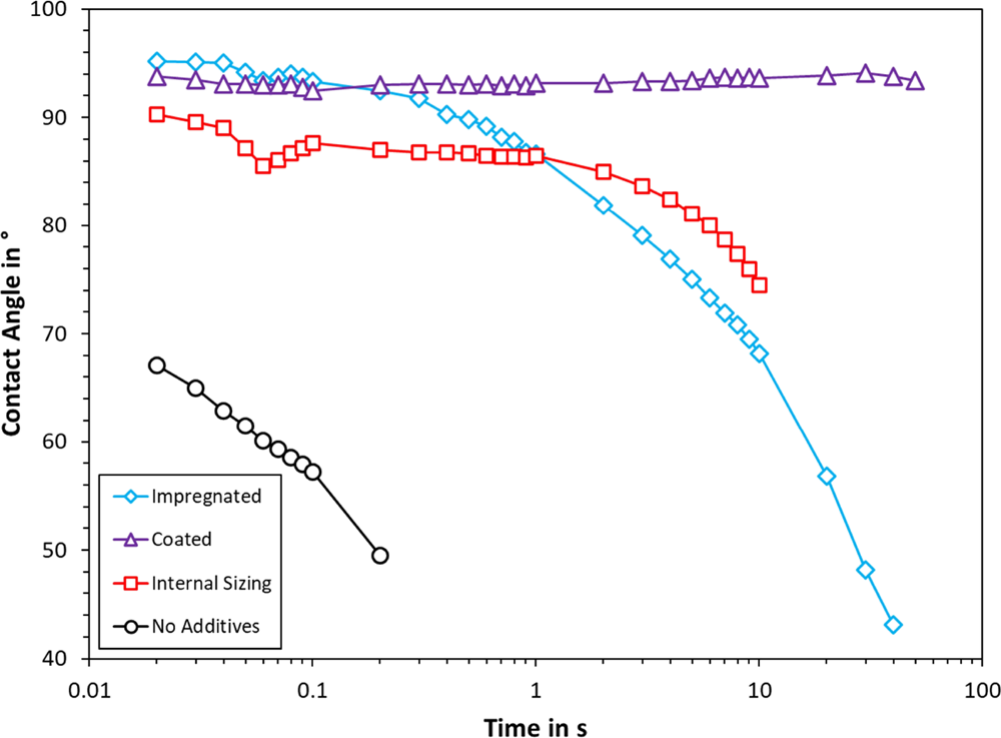
Effect of application mode on the contact angle of handsheets thermoformed at 150 °C and 45 bar. In each case, organosolv lignin was added to bleached CTMP. Each graph is the average of in total 20 measurements.

**Figure 6:**
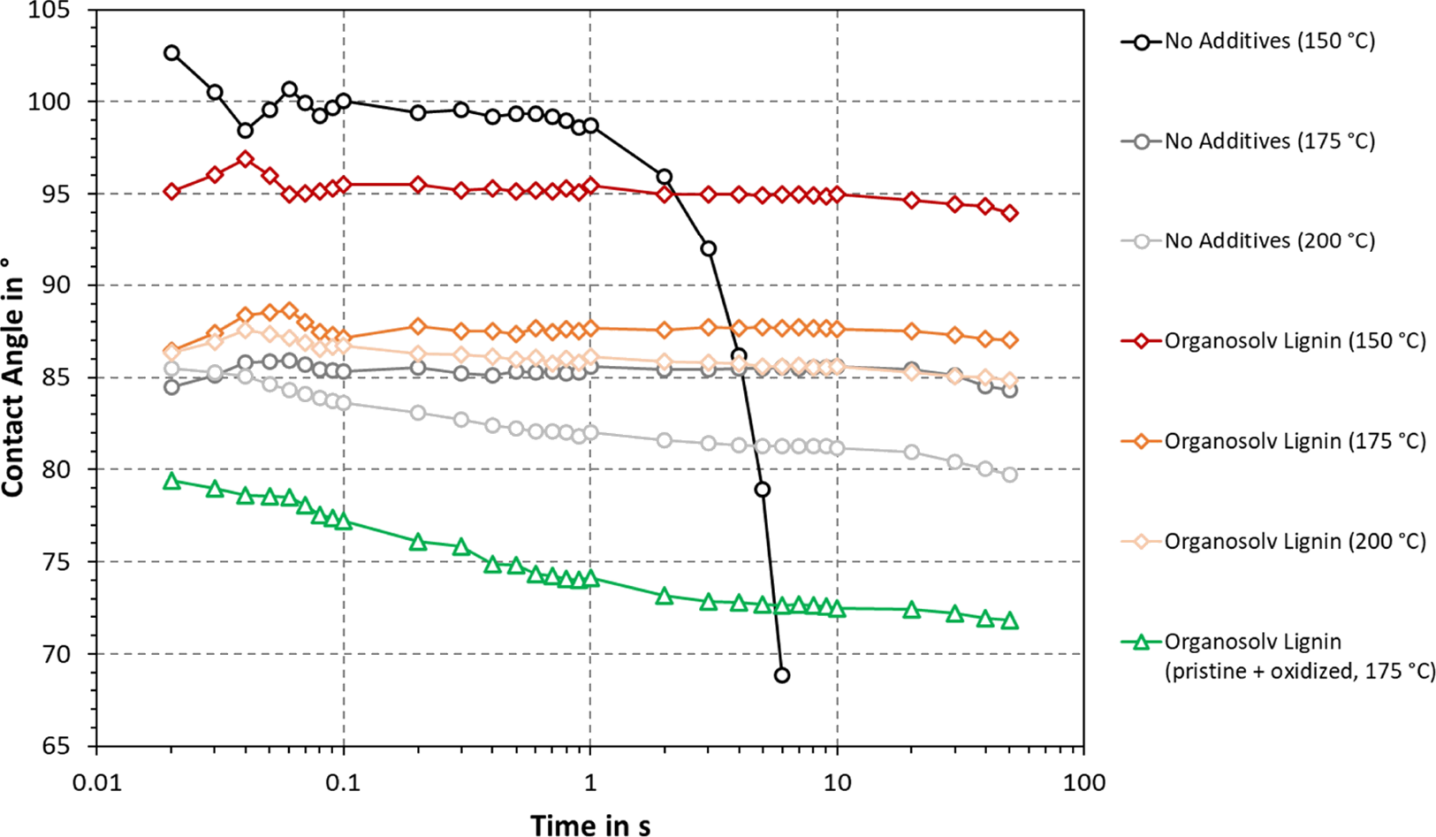
Effect of temperature and lignin additives on contact angle of handsheets thermoformed at 500 bar. In each case, the organosolv lignin was added to unbleached CTMP. Each graph is the average of in total 20 measurements.

**Figure 7:**
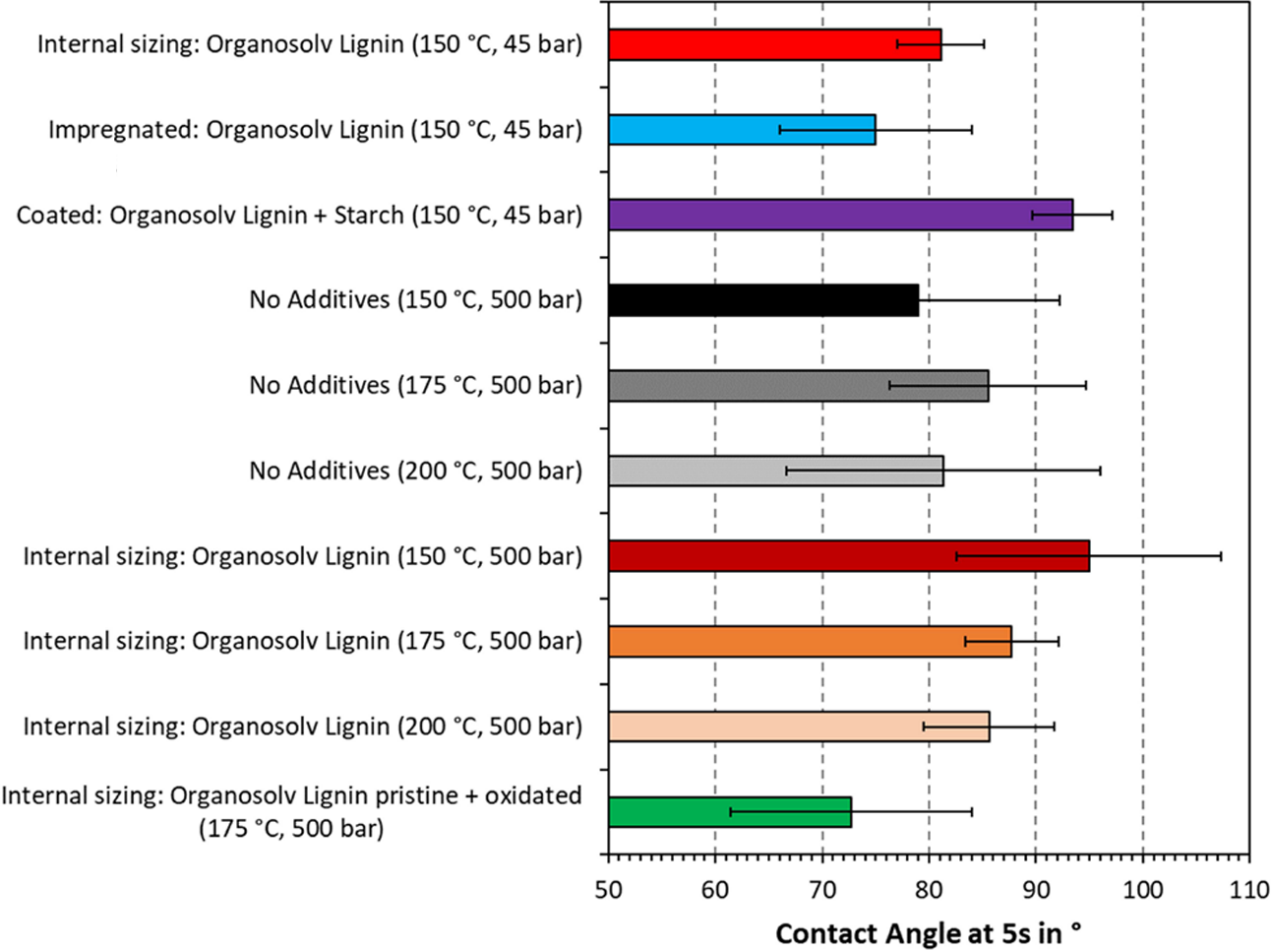
Contact angle after 5 s of handsheets thermoformed with bleached (45 bar) or unbleached CTMP (500 bar) and various additives. Each graph is the average of 20 measurements with standard deviation indicated as error bars.

The time-dependent data of contact angle measurements are plotted in Figures 5 and 6. For comparison, the contact angles at 5 s are also displayed in Figure 7. As can be seen in Figure 5, the bleached CTMP pressed at 45 bar exhibited the lowest contact angle. The measurement ceased after less than a second, implying that water was quickly absorbed by the fiber material. Unbleached CTMP pressed at 500 bar and the same temperature (150 °C) also absorbed the droplet within a few seconds; however, the initial contact angle was larger and droplet absorption took more than 10 times longer. The CSF of both substrates is the same, and the ISO difference of unbleached CTMP is lower. Bleaching involves chemical modification of the fiber surface and limited removal of lignin. Based on their production, the unbleached CTMP would hence be the less hydrophilic substrate of the two. In our previous study, we indeed found that unbleached CTMP had a lower water uptake than bleached CTMP;^12^ however, this difference was only 15%. The greater densification is hence a better explanation for the observed difference in contact angle, that is, unbleached CTMP at 728 kg/m^3^ compared to bleached CTMP at 307 kg/m^3^, which were pressed at 500 and 45 bar, respectively (Table 3).

Figure 5 furthermore compares the effect of application mode on contact angle. The contact angle is a measure of the hydrophobicity of the material. A surface is considered hydrophobic when the contact angle is greater than 90 °C. Here, impregnation with organosolv lignin yielded a higher initial contact angle than internal sizing. However, the contact angle of the impregnated substrate fell below that of internal sizing after 1 s. Both application modes yielded the same basis weight at 177–178 g/m^2^ and should hence contain the same percentage of added lignin. It appeared that internal sizing was more favorable in reducing long-term wetting and was hence used in subsequent experiments. Coating with organosolv lignin and starch also showed great potential, yielding a constant contact angle of 93°. Such blends may also provide superior barrier properties.

Three different temperature settings were compared in Figure 6. The initial contact angle was the highest for the two samples thermoformed at 150 °C. The contact angle is, among others, affected by the chemical make-up and surface roughness. As mentioned in the discussion of Figure 4, the surface roughness appeared higher for handsheets pressed at 150 °C, which is in agreement with our results in Figure 6. In analogy to this, the contact angle consistently decreased with increasing thermoforming temperatures. Changes in the material chemistry may further contribute to surface smoothing, in particular at high temperatures such as 200 °C. At 175 and 200 °C, the effect of the added organosolv lignin was an increase in contact angle by 2–5°. At 150 °C, the contact angle was initially higher for unbleached CTMP without added lignin; however, after enough time (3 s), the contact angle was higher for the sample with added lignin. The idea of blending oxidized and pristine organosolv lignins was to introduce a cross-linker as our previous study showed that chemical reactions can occur during thermoforming.^12^ In this implementation, it appeared that adding oxidized organosolv lignin yielded a slight decrease in density (Table 3) compared to the sample containing only pristine organosolv lignin. The observed contact angle in Figure 6 was also lower. These results could be explained by the lower ability to fill cavities within the fiber network. In addition, oxidation is known to introduce carboxylation, which would increase the hydrophilicity of the material.

As the general trends have been outlined in the discussion so far, Figure 7 will be primarily used to discuss the experimental error. The standard deviation is indicated by the error bars, showing a deviation of 3–15°. This corresponds to a relative error of 4–18%, which is high compared to other methods. Such data scattering is frequently observed in contact angle measurements and was hence anticipated by measuring 20 data points per sample. The observed trends are therefore still deemed statistically significant.

Contact angle measurements consider only one-dimensional uptake of water, that is, orthogonal to the plane of the material. This testing method is widely established as it enables the assessment of surface modifications, coatings, and other characteristics. Contact angle measurements on pulp and paper products are kinetic as the fibers are wetted during the measurement. This measurement is hence only an instantaneous representation. The water uptake was therefore measured in addition as this provides a measure of equilibrium rather than kinetics. Experimentally, the handsheets were cut into short stripes which were immersed in water for 24 h. Water can penetrate from all three dimensions, providing a close representation of the water content in the saturated state.

**Figure 8:**
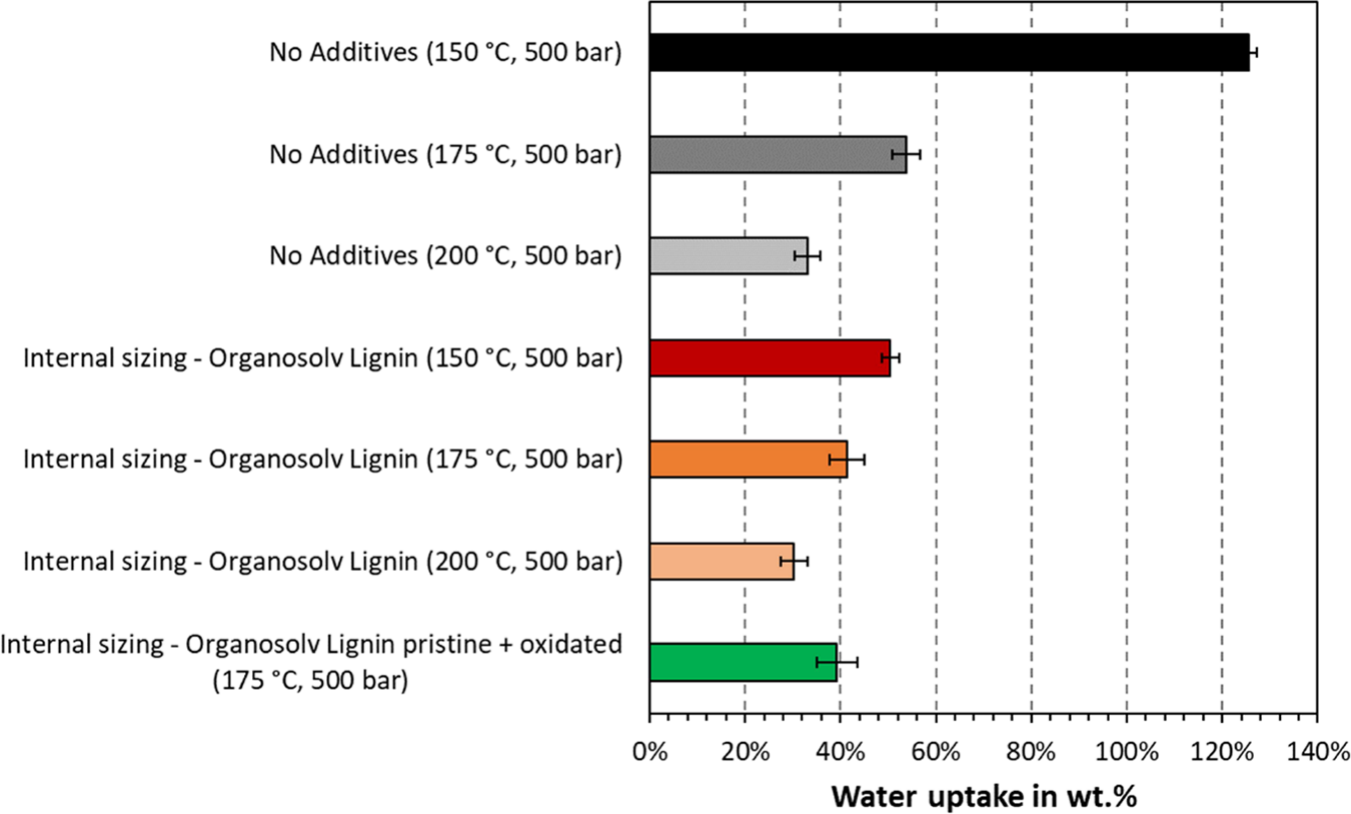
Water uptake of unbleached CTMP handsheets with and without added organosolv lignin. Each graph is the average of three measurements with standard deviation indicated as error bars.

The water uptake of samples thermoformed at 500 bar is depicted in Figure 8. A general trend is found where increasing temperatures yielded a lower water uptake for each sample composition. Addition of organosolv lignin reduced the water uptake compared to the blank sample. This difference is greatest for samples thermoformed at 150 °C, that is, unbleached CTMP exhibited a water uptake of 125 wt % without and 50 wt % with organosolv lignin, respectively. It is interesting to note that addition of oxidized organosolv lignin yielded the same water uptake as the unmodified lignin alone. Furthermore, the overall trend in Figure 8 is dissimilar to the trend in Figure 5. The contact angle showed a decreasing tendency with increasing temperatures, which was opposite for the water uptake. As discussed, the contact angle was likely higher at lower temperatures due to a higher surface roughness. The ability of the sample to take up water, on the other hand, seemed to decrease at higher temperatures and after adding organosolv lignin. The latter is in agreement with the contact angle measurements as samples with added organosolv lignin exhibited higher contact angles after 3 s than the blank samples. The long-term ability to resist wetting may hence be related to the lower water uptake of the samples. Still, our results show that there are multiple effects governing the action of added lignin.

### Microscope Images

**Figure 9:**
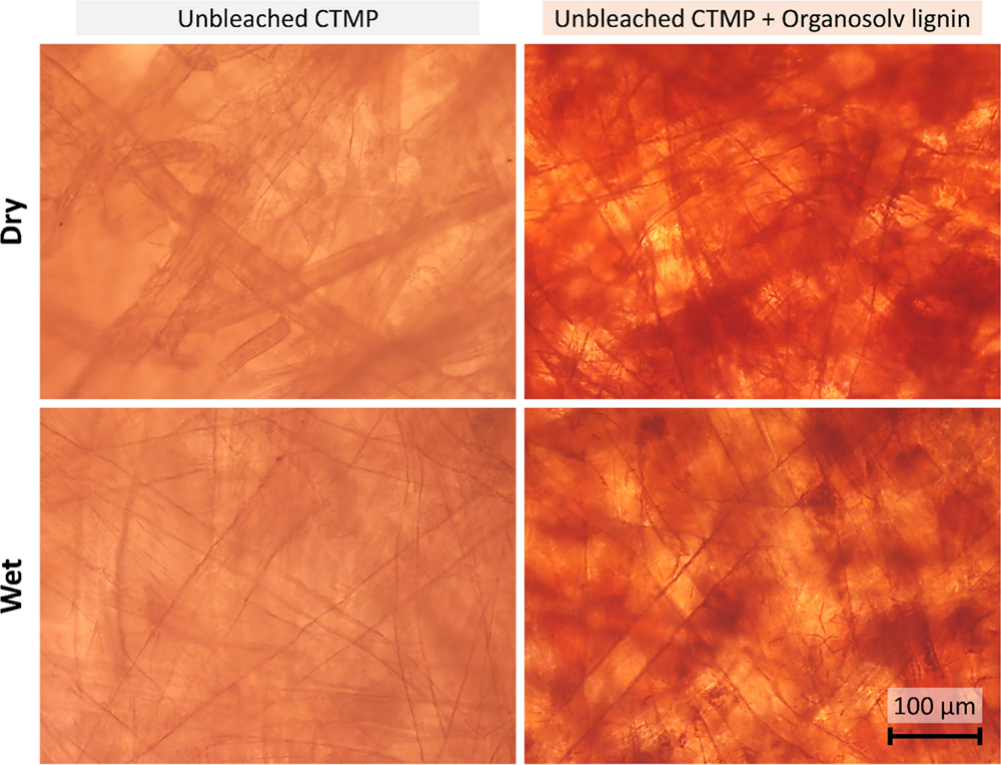
Light microscope images of unbleached CTMP (left) with organosolv lignin (right) thermoformed at 150 °C and 500 bar. Images at the top were recorded in an air-dry state, whereas those at the bottom were taken after immersion in water for 24 h. All images were contrast- and brightness-adjusted for better visibility.

To further study the mechanism that governs the effect of added lignin, light microscope images of dry and wet handsheets were recorded. The two samples are exemplarily shown in Figure 9, where the fibers are visible as dark cylindrical shadows within the sample. As can be seen, the fiber thickness is greater in the wet state. The same procedure for wetting was used as for measuring the water uptake, that is, immersion in water for 24 h. The swelling was greater for the sample without added lignin. This might appear trivial at first as the water uptake was greater without added lignin. However, it also elucidates the functioning mechanism of the added lignin, which is restricted swelling of the fibers. The same trend was also observed for samples thermoformed at higher temperatures, that is, 175 and 200 °C. Overall, reduced swelling does not rule out other effects such as the added lignin filling cavities that could take up water via capillary forces. Adding lignin may also affect mass transfer by providing a denser structure. The latter is not considered a governing effect for water uptake measurements as the samples were equilibrated at the measurement instance (no net mass transfer).

**Figure 10:**
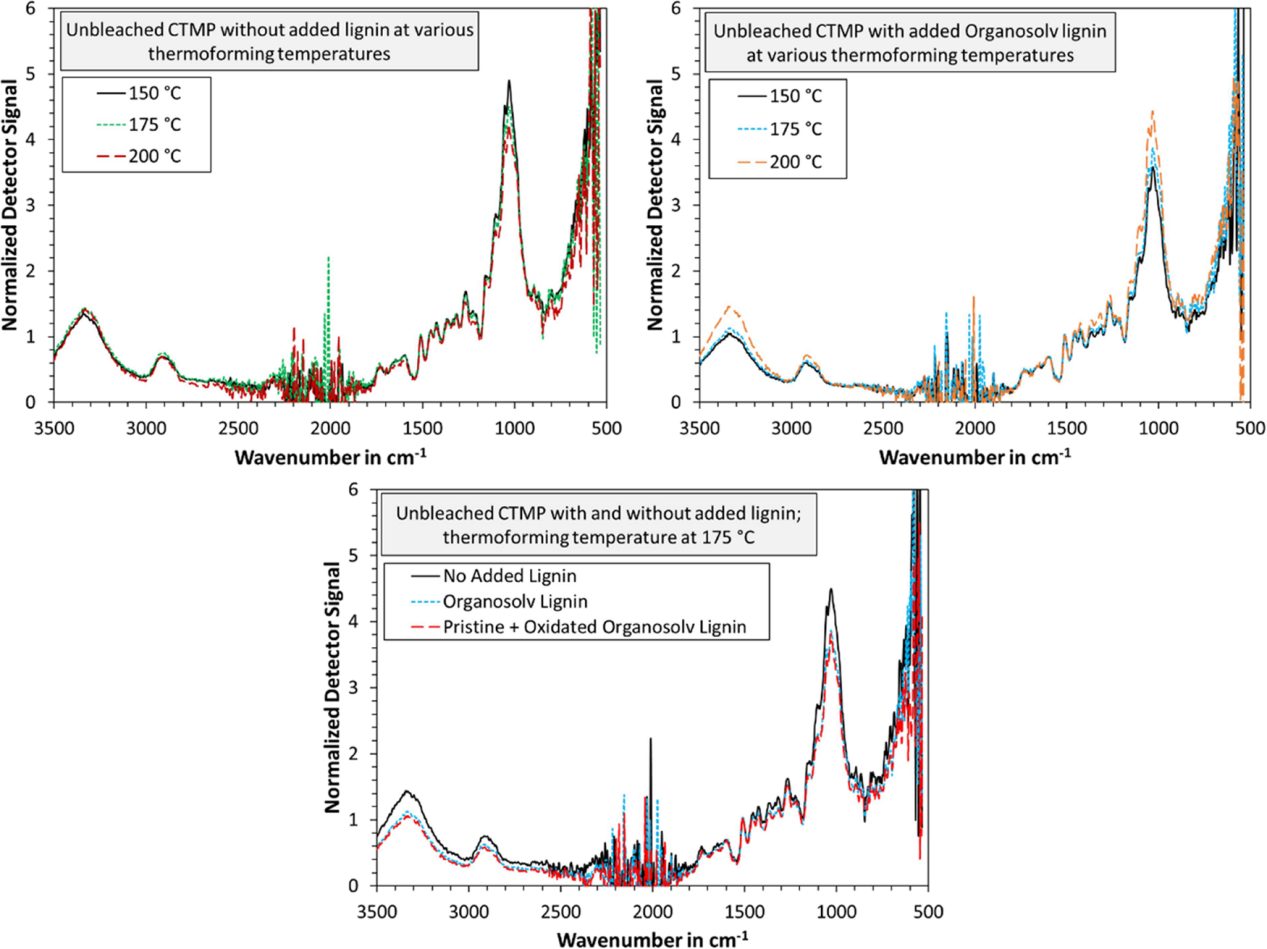
ATR–FTIR graphs of thermoformed handsheets with and without added lignin. Each graph was baseline-corrected and normalized via the aromatic skeletal vibrations at 1505–1510 cm^–1^.

At last, FTIR measurements of the thermoformed handsheets were performed. The data are plotted in Figure 10 using baseline correction and normalization at the aromatic skeletal vibrations (1505–1510 cm^–1^). As can be seen, the differences are minor. Little to no change was observed due to increasing temperatures for the blank samples. Adding organosolv lignin decreased the observed amount of hydroxyl groups (OH stretching at 3412–3460 cm^–1^); however, this effect could likely be due to more organosolv lignin being present at the fiber surface, which has fewer OH groups than cellulose. Increasing the temperature of samples with added lignin also increased the OH stretching band, which is likely due to more cellulose being present at the surface. If reactions between the added lignin and OH groups of the cellulose were to take place, this should in theory yield a decrease of the OH stretching band. However, the opposite of this was observed, indicating that increasing the thermoforming temperature facilitated the flow of organosolv lignin into the cavities within the fiber network. No change in the absorption band at 1700–1730 cm^–1^ (ester band) was detected, which had been observed in our previous study.^12^ We hence conclude that there was no cross-linking between the added lignin and the CTMP fibers.

## Conclusions

In this study, the effect of adding organosolv lignin as an internal sizing additive to thermoformed pulp materials and handsheets was investigated. Knowledge on heterogeneity and complexity of the lignin structure and chemical reactivity after the isolation process is important for lignin valorization. Compared to other technical lignin available in the market, organosolv lignin has a low molecular weight, is sulfur-free, has a low ash content, is more hydrophobic, and has a lower *T*_g_.

When organosolv lignin was added as an internal sizing agent to the thermoformed pulp products, the water uptake and swelling of fibers were reduced. The effects were more pronounced at higher thermoforming temperatures and high pressures. The added lignin may act as a binder within the fiber network, which can reduce the dimensional expansion of the fibers. The ability of the entire material to take up water was hence reduced, improving the overall water resistance. However, the contact angle was highest when lignin was used as an internal sizing agent to the thermoformed pulp products formed at the lowest temperature (150 °C) and a high pressure. The organosolv lignin is inherently less hydrophilic than the fiber material, hence resulting in higher long-term contact angles and lower water uptake.

Another interesting result was that if organosolv lignin was applied as a coating additive together with starch, an increase in water resistance and a high contact angle were observed. This is a promising property especially when the aim is to use the thermoformed products to packaging applications. In comparison with other technical lignin studied in our previous study,^12^ we found a greater effect of organosolv lignin on hydrophobization and reduced wetting of thermoformed pulp products. These properties render great potential to organosolv lignin as a functional sizing agent for thermoformed pulp materials.

Overall, the results from our study show the possibility of using organosolv lignin as an internal sizing additive to thermoformed pulp products with improved water resistance, reduced fiber swelling, and increased contact angles. As such, these thermoformed products provide an alternative solution to replace the current petrochemical nonbiobased products used for a wide range of packaging applications, which may in addition have a favorable impact on the economy of sustainable biorefineries.

## Abbreviations

CTMP: chemi-thermomecahnical pulp
ATR–FTIR: attenuated total reflectance–Fourier transform infrared spectroscopy
TGA–DSC: thermogravimetric analysis coupled with differential scanning calorimetry
SEC: size-exclusion chromatography
CSF: Canadian standard freeness
DMSO: dimethyl sulfoxide
DM: dry matter
*T*_g_: glass transition temperature
